# Neural mechanism of binding amplitude information of echo sound with its frequency one in echolocating bat

**DOI:** 10.1186/1471-2202-13-S1-P82

**Published:** 2012-07-16

**Authors:** Yoshitaka Mutoh, Yoshiki Kashimori

**Affiliations:** 1 Department of Engineering Science, University of Electro-Communications, Chofu, Tokyo 182-8585, Japan; 2 Graduate School of Information Systems, University of Electro-Communications, Chofu, Tokyo, 182-8585, Japan

## 

Most species of bats making echolocation use the sound pressure level (SPL) and Doppler-shifted frequency of ultrasonic echo pulse to measure the size and velocity of target. The neural circuits for detecting these target features are specialized for amplitude and frequency analysis of the second harmonic constant frequency (CF2) component of Doppler-shifted echoes. The neuronal circuits involved in detecting these echo features have been well known [1]. In natural situation, large natural objects in environment, like bushes or trees, produce complex stochastic echoes, which can be characterized by the echo roughness. The echo signal reflecting from a target insect is embedded in the complex signal. Even in such an environment, bats can detect accurately the detailed information of flying insect. We consider here two questions: how the neural circuits bind amplitude and frequency information of echo sound, and how bat distinguishes target information from information of background signal. To address these issues, we developed a neural network model for detecting SPL amplitude and Doppler-shifted frequency of echo sound.

The model contains two hemispheres, in each of which, the network model consists of cochlea (Ch), inferior colliculus (IC), and Doppler-shifted constant frequency (DSCF) processing area. The Ch network has a frequency map by which sound frequency is encoded. The model for detecting frequency information of echo sound was based on the model previously presented by us [2].The SPL amplitude is encoded in to firing rate of IC neurons. The IC neurons encode SPL amplitude by means of a balance between excitatory connection from contralateral Ch neurons and inhibitory connection from ipsilateral ones, and then combine the amplitude and frequency information of echo sound. The DSCF network has two types of sub-networks detecting AC and DC components of echo sound, which represent the information of target and background signals, respectively.

We showed that in IC, the amplitude information of echo sound is encoded by integrating the outputs of ipsi and contralateral Ch neurons and then combined with the Doppler-shifted frequency information encoded by tonotopical map of Ch neurons. The accuracy of the amplitude and frequency information was improved in the DSCF area. The model reproduced well several experimental results observed in IC and DSCF neurons. We also showed that AC and DC components of echo signal are discriminated in the two sub-networks of DSCF. The discrimination ability is due to the difference in time constant between DSCF neurons in the two sub-networks.

## Conclusion

We presented the neural mechanism for detecting SPL amplitude and Doppler-shifted frequency of echo sounds. The model offered the neural mechanism of how amplitude and frequency information of echo sound are processed and then combined in IC and DSCF area. We also presented that DSCF area discriminates between AC and DC components of echo sound.
